# Detecting protein complexes in protein interaction networks using a ranking algorithm with a refined merging procedure

**DOI:** 10.1186/1471-2105-15-204

**Published:** 2014-06-19

**Authors:** Eileen Marie Hanna, Nazar Zaki

**Affiliations:** 1 College of Information Technology, United Arab Emirates University (UAEU, Al Ain 17551, United Arab Emirates

**Keywords:** Google pagerank algorithm, PPI, Protein complex, Essential protein, ProRank algorithm

## Abstract

**Background:**

Developing suitable methods for the identification of protein complexes remains an active research area. It is important since it allows better understanding of cellular functions as well as malfunctions and it consequently leads to producing more effective cures for diseases. In this context, various computational approaches were introduced to complement high-throughput experimental methods which typically involve large datasets, are expensive in terms of time and cost, and are usually subject to spurious interactions.

**Results:**

In this paper, we propose ProRank+, a method which detects protein complexes in protein interaction networks. The presented approach is mainly based on a ranking algorithm which sorts proteins according to their importance in the interaction network, and a merging procedure which refines the detected complexes in terms of their protein members. ProRank + was compared to several state-of-the-art approaches in order to show its effectiveness. It was able to detect more protein complexes with higher quality scores.

**Conclusions:**

The experimental results achieved by ProRank + show its ability to detect protein complexes in protein interaction networks. Eventually, the method could potentially identify previously-undiscovered protein complexes.

The datasets and source codes are freely available for academic purposes at http://faculty.uaeu.ac.ae/nzaki/Research.htm.

## Background

Proteins often collaborate by forming groups referred to as protein complexes in order to execute various cellular functions [1]. Accordingly, identifying protein complexes in protein interaction networks is an essential step towards better understanding normal and abnormal cellular processes. The higher the amounts of discovered biological information, the greater are the possibilities to design more effective medical treatments for numerous diseases. The biological methods employed for the detection of protein complexes often face drawbacks, mainly in time and cost requirements. Therefore, many computational methods were designed in order to complement the experimental efforts; for instance, by highlighting protein groups which could potentially delineate various cellular functions. In a computational context, a protein interaction network is usually modeled as an interaction graph in which vertices represent the proteins and edges represent their interactions. In this setting, it is generally assumed that protein complexes correspond to dense subgraphs. Among the recent methods, we herein highlight: Markov Clustering (MCL) [2] which uses random walks in protein interaction networks, the molecular complex detection (MCODE) algorithm [3] which identifies complexes as dense regions grown from highly-weighted vertices, the clustering based on maximal cliques (CMC) method [4], the Affinity Propagation (AP) algorithm [5], ClusterONE [6] which identifies protein complexes through clustering with overlapping neighborhood expansion, the restricted neighborhood search (RNSC) algorithm [7,8], the RRW algorithm which generates complexes by using repeated random walks [9], CFinder [10] which is based on the clique percolation method and the GIBA tool [11] which consists of clustering and filtering steps to generate the set of protein complexes corresponding to a given protein interaction dataset. These methods, among several ones, showed relatively good performance in detecting protein complexes. However, the assumption that protein complexes correspond to dense subgraphs in the interaction network limits the detection process because it does not usually allow the identification of complexes with few members and/or few interactions. ProRank [12] is a recent method developed to detect protein complexes from protein interaction networks based on a protein ranking algorithm. When compared with previous methods, the experimental studies showed better results for the ProRank algorithm in terms of the number of detected protein complexes as well as precision, recall and accuracy levels. In spite of that, ProRank does not take into account possible overlaps among the detected complexes. In fact, a protein can exhibit many functions by being part of different complexes [13]. Therefore, it is indeed beneficial to reflect this fact when searching for protein complexes in interaction networks. Moreover, ProRank computes a similarity matrix consisting of the similarity scores among all the proteins in the network. This step can be discarded since it is computationally-expensive and has a comparatively small effect on the final results [14]. In this paper, we present ProRank+, an enhanced protein-complex detection algorithm which is able to detect possibly-overlapping complexes. Additionally, the method includes a novel merging procedure, *Merging by Cohesiveness*, used to refine the detected protein complexes. Here, complexes are viewed as entities of highly-interconnected members that are well-separated from the rest of the interaction network. The experimental studies and results greatly favor our approach.

## Methods

### The ProRank method

**Figure 1:**
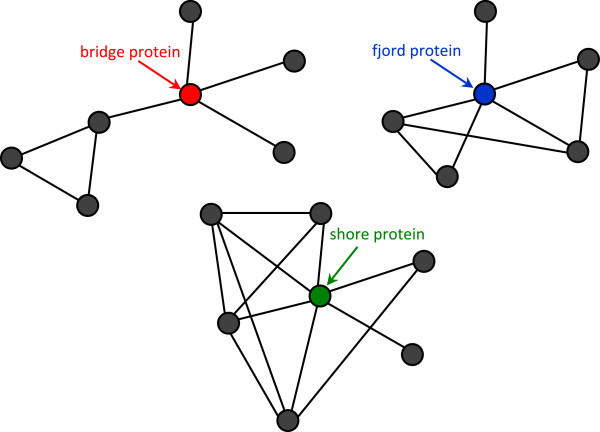
Examples of bridge, fjord and shore proteins in protein-protein interaction networks.

ProRank [12,14] is a recent protein complex-detection method. It mainly consists of a protein ranking algorithm inspired by Google’s PageRank algorithm [15-18] which quantifies and ranks web pages according to their level of importance. Likewise, ProRank applies the same analogy on protein interaction networks to rank proteins in interaction networks and thus pinpoint the “essential” ones which most-likely play key roles in cellular functions. Those proteins could then be considered as starting points based on which the detected complexes can be formed. In addition, the method also takes into account the evolutionary relationships among protein members of the same complex by calculating their pairwise similarities. Five main steps delineate the ProRank algorithm:

1. Pruning: removing unreliable interactions which could negatively affect the detection process using the AdjustCD method [19,20], a weighting scheme that iteratively calculates the reliability of protein interactions based on the topology of the interaction network. Then, the interactions with scores less than a specified threshold are discarded.2. Filtering: a protein interaction network usually contains noisy proteins which may belong to one of three defined types: bridge proteins which have a disconnected subgraph of neighbors; fjord proteins whose neighbors have a small number of interactions among each other; and shore proteins which have at least one neighbor with significantly few interactions with other proteins. Accordingly, proteins in the network are examined for potential memberships in these types. Figure 1 illustrates examples of the described categories.

3. Protein Similarity Calculating: proteins belonging to the same complex most likely have evolutionary relationships. Consequently, the similarity scores among all the proteins in the network are calculated using pairwise alignment.

4. Protein Ranking: a ranking algorithm, analogous to the PageRank algorithm, is used to order proteins based on two criteria: the number of interactions in which they participate and their similarity levels with other proteins.

5. Complex Detection: using the spoke model, essential proteins, which do not belong to any of the types defined in step 2, are consecutively considered by their decreasing ranking order and each of them is pulled from the interaction network along with its neighbors to form a protein complex. Here, each protein can belong to one complex only.

**Figure 2:**
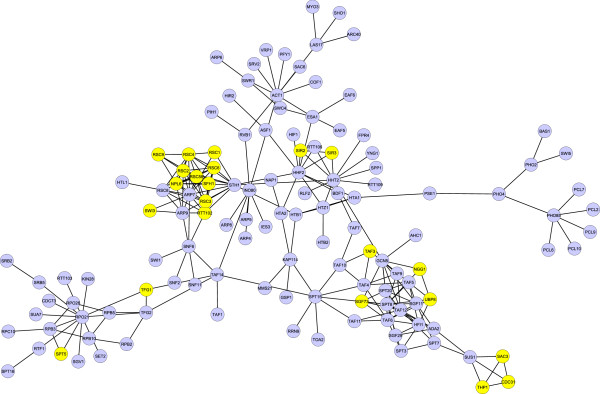
**A yeast protein-protein interaction sub-network.** The nodes coloured in yellow correspond to essential proteins identified by the ProRank algorithm.

**Figure 3:**
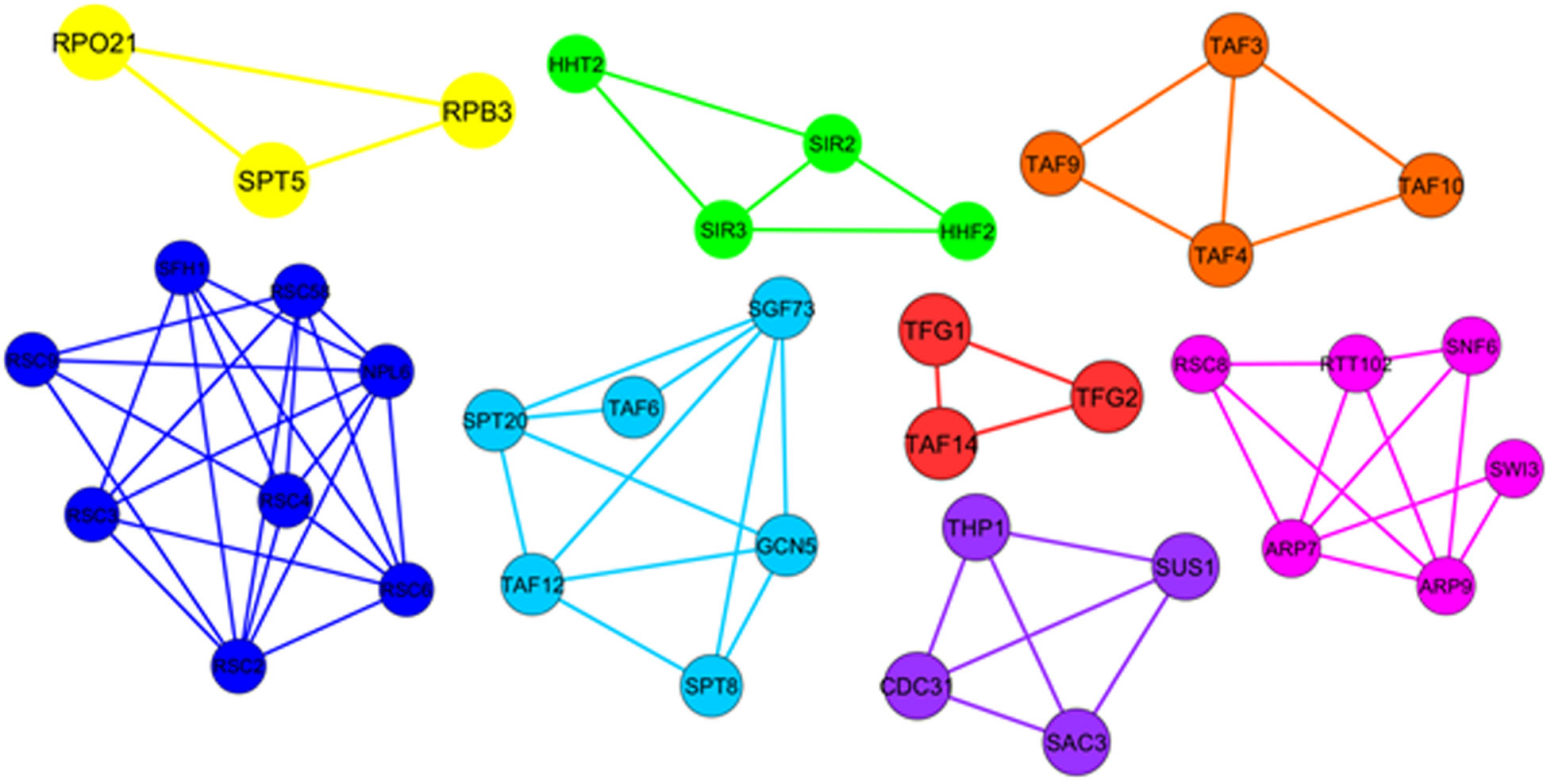
**The detected complexes by the original ProRank algorithm when applied on the sub-network in Figure **2**.**

In addition to the steps mentioned above, the ProRank algorithm discards formed protein complexes of less than three members. Also, it merges two complexes if more than 50% of the neighbors of each protein belonging to the first complex are in the second complex. To show the potential of the approach, we consider the network presented in Figure 2. It is a sub-network generated from the yeast protein-protein interaction dataset at the Mentha interactome browser [21], version date 05/01/2014. The sub-network includes of 235 interactions. It corresponds to the largest connected portion of the network consisting of proteins which participate in the interactions of scores greater than or equal to 0.99, and their inter-connections of scores greater than or equal to 0.8. The nodes colored in yellow highlight the essential proteins identified by ProRank and the resulting protein complexes are presented in the first row of Figure 3.

### The ProRank + method

Although the ProRank method achieved competitive results when compared to other approaches, it could still be improved further. Pruning, filtering, ranking and complex-detection steps constitute strong building blocks of the approach. This is true since unreliable interactions can be removed by the pruning step; noisy proteins can be identified by the filtering phase; essential proteins can be ordered based on their importance in the network by the ranking step; and detected complexes can be formed starting from essential proteins by the spoke model at the complex-detection stage. On the other hand, the similarity calculating step by which the similarities among all the proteins in a given interaction network are computed is computationally very expensive because such networks are typically very large. Since the effect of this step on the final results is relatively minimal [12] and because the high similarity among proteins is not exclusive to the ones belonging to the same complex, it can be excluded. Consequently, this will decrease the required computation time and will not compromise the quality of the final results.

**Figure 4:**
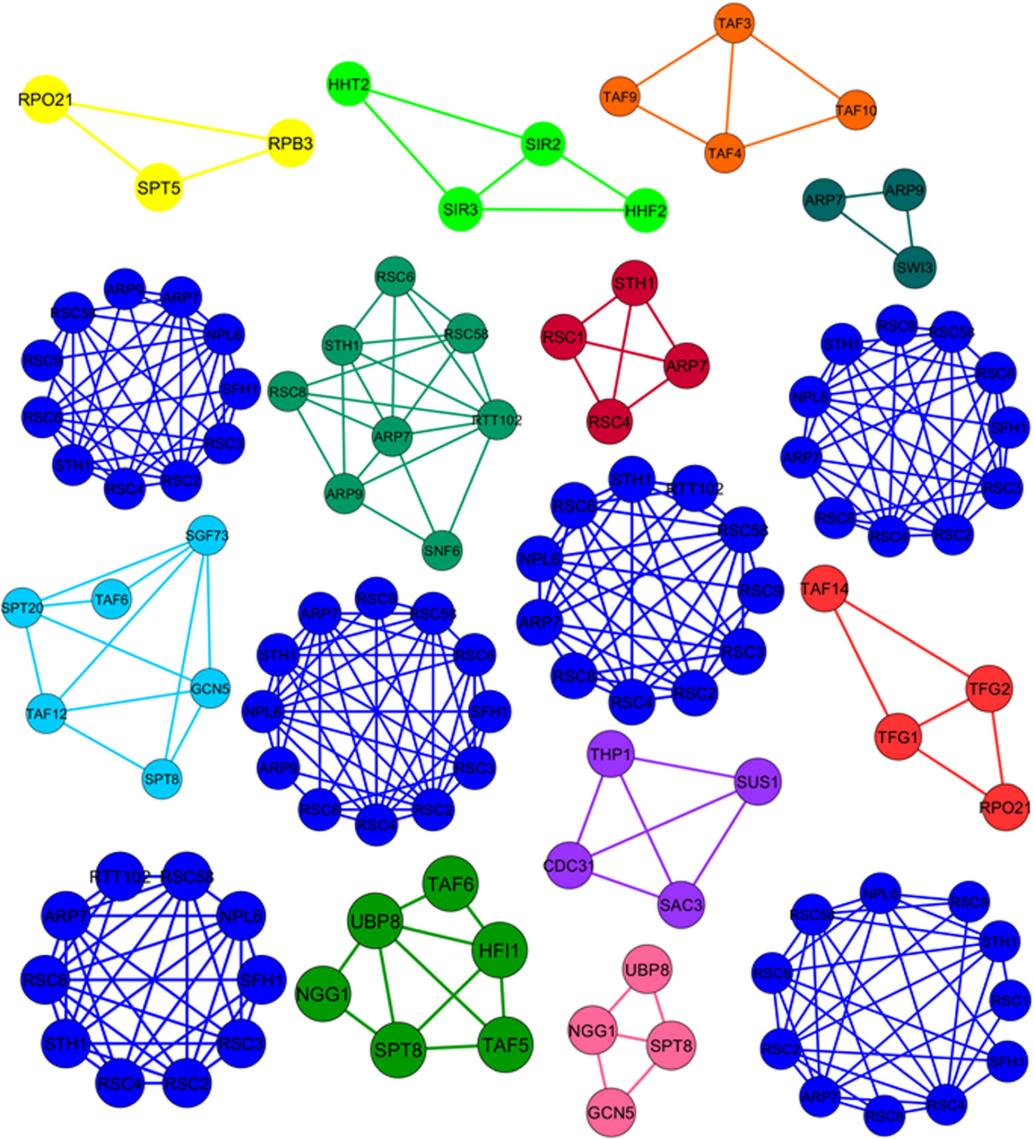
**The detected complexes by the ProRank algorithm with the complex-overlap assumption when applied on the sub-network in Figure **2**.**

**Figure 5:**
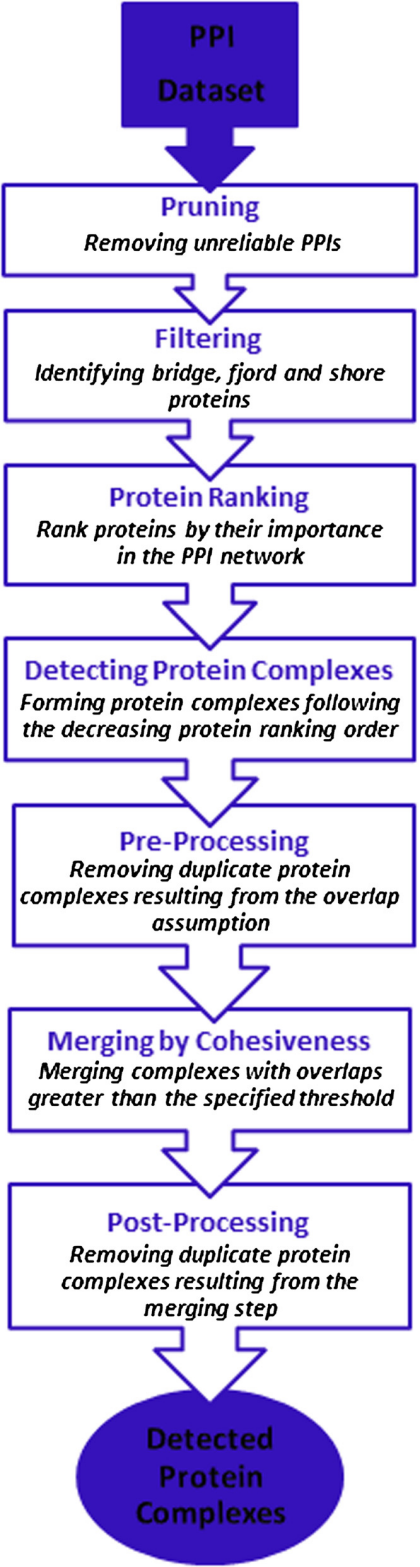
A schema representing the steps which delineate the ProRank + algorithm.

Added to that, proteins could contribute in multiple cellular functions by being part of several protein complexes [13]. For instance, among the 1189 proteins contained in the MIPS catalog of protein complexes [22], 820 proteins (approx. 69%) belong to more than one complex. Similarly, among the 1279 covered by the SGD complex set [23], 332 proteins (approx. 26%) belong to multiple complexes. A protein interaction network is hence expected to comprise overlapping complexes, and accounting for this biological fact would most likely lead to more accurate complex-detection results. Accordingly, let us observe the effect of this adjustment on the protein-protein interaction network presented in Figure 2. The detected complexes, corresponding to applying the ProRank method with the added overlap assumption, are listed Figure 4. The results uphold the improvement added by the overlap extension which could potentially lead to a more correct detection of protein complexes. Actually, by allowing proteins to belong to more than one complex, the number of complexes formed from the identified essential proteins becomes higher indeed. However, it can be noticed that the amount of overlaps among some the detected complexes is relatively high. This was anticipated. Actually, since all essential proteins are now seeds for forming protein complexes, the ones that share numerous neighbors will certainly produce close and highly-overlapping protein complexes. In order to overcome this limitation and to further improve the quality of the predicted complexes, the following filtering and merging steps are added to the algorithm (Figure 5):

1. The set of generated complexes is filtered to remove duplicates resulting from the complex-overlap notion.

2. Next, a merging procedure referred to as *Merging by Cohesiveness*, is applied in the direction of exploring more variations of the detected complexes. In consistency with the initial considerations of the ProRank method, we rely on the key roles of the essential proteins in the network to establish the merging process. All the detected complexes are matched against each other for possible merging. Two complexes, *C1* and *C2*, whose percentage of overlapping essential proteins is above a merging threshold, *merging_threshold*, are merged along with their interconnections to form a larger complex *C*. Then, the process adopts the cohesiveness measure introduced in [6] to assess the quality of the resulting complex and its iteratively-extended variants defined hereafter. The cohesiveness of a complex *C* is given by equation (1):

(1) $$\text{Cohesive}\left(C\right)=\frac{w_{\mathrm{in}}\left(C\right)}{w_{\mathrm{in}}\left(C\right)+w_{\mathrm{out}}\left(C\right)+p}$$

where *w*_*in*_*(C)* is the sum of the weights of edges that are entirely contained in *C*, *w*_*out*_*(C)* is the sum of the weights of edges that connect the proteins belonging to *C* to the rest of the network, and *p* is a penalty term reflecting uncertainties in the protein interaction network. This cohesiveness measure was developed to model the assumption by which a protein complex is viewed as an entity with strongly-interconnected members that is well-separated from the rest of the network. The successive steps of our merging procedure aim at refining merged complex while increasing their cohesiveness measures. For each protein, *prot*, contained in *C*: first, the set of its neighbors, *N*_*prot*_, is formed; then, for each neighbor protein, *n*_*prot*_, in *N*_*prot*_, the complex *C’ = C ∪ {n*_*prot*_*}* is constructed; and if the cohesiveness of *C’* is greater or equal to the cohesiveness of *C*, *n*_*prot*_ is added to *C*. After exploring all the proteins initially belonging to *C* in the same manner, the derived complex is added to the final list of detected complexes. The pseudocode of merging two complexes, *Merge_by_Cohesiveness*, is presented in next sub-section.

3. Possible duplicate complexes may also result from the merging processes. Therefore, an additional duplicate-filtering step is added in order to ensure the accuracy of the results.

## Results and discussion

### Datasets and evaluation criteria

ProRank + was tested on five large-scale protein-protein interaction datasets associated to the well-studied yeast microorganism. Four of the datasets consist of weighted protein interactions, they are: Collins [24], Krogan core and Krogan extended [25], and Gavin [1]. The fifth dataset, BioGRID [26], contains unweighted interactions. The properties of the 5 datasets used in the experimental work are shown in Table 1. The set of predicted complexes was matched against the MIPS catalog of protein complexes [27]. The datasets and the reference set of complexes were used to evaluate the ClusterONE method and to compare its performance with other approaches. We also adopted the same quality scores applied in [6] to assess the quality of our algorithm. In addition, it is important to note that the parameters of the compared algorithms were optimized in such a way to produce best possible results. The quality scores cover: (a) the number of complexes in the reference catalog that are matched with at least one of the predicted complexes with an overlap score, *w*, greater than 0.25; (b) the clustering-wise sensitivity (*S*_*n*_); and (c) the clustering-wise positive predictive value (*PPV*) which were originally introduced in by Brohée and van Helden in [28] to calculate the matching quality, mainly in terms of the correctly-matched protein members among the detected complexes; (d) the geometric accuracy (*Acc*) which is the geometric mean of *S*_*n*_ and *PPV*; and (e) the maximum matching ratio (*MMR*) which reflects how accurately the predicted complexes represent the reference complexes by dividing the total weight of the maximum matching by the number of reference complexes. Given *m* predicted complexes and *n* references complexes, and based on the confusion matrix, *T = [t*_*ij*_*]*, the corresponding formulae are given by the following equations ((2,3,4,5)) where *t*_*ij*_ represents the number of proteins that are found in both predicted complex *m* and reference complex *n*.

**Table 1:** The properties of the five datasets used in the experimental study

**Dataset**	**Proteins**	**Interactions**	**Network density**	**Average no. of neighbors**
**Collins**	1,622	9,074	0.007	11.189
**Krogan core**	2,708	7,123	0.002	5.261
**Krogan extended**	3,672	14,317	0.002	7.798
**Gavin**	1,855	7,669	0.004	8.268
**BioGRID**	5,640	59,748	0.004	21.187

(2) $$w\left(A,B\right)=\frac{{\left|A\cap B\right|}^{2}}{\left|A\right|\left|B\right|}\;$$

(3) $$S_{n}=\frac{\sum_{i=1}^{n}\mathrm{ma}x_{j=1}^{m}t_{\mathrm{ij}}}{\sum_{i=1}^{n}n_{i}}$$

(4) $$\mathrm{PPV}=\frac{\sum_{j=1}^{m}\mathrm{ma}x_{i=1}^{n}t_{\mathrm{ij}}}{\sum_{j=1}^{m}\sum_{i=1}^{n}t_{\mathrm{ij}}}$$

(5) $$\mathrm{Acc}=\sqrt{S_{n}\times \mathrm{PPV}}$$

### Experimental settings of ProRank+

The steps of applying ProRank + on a given dataset, *D*, and their experimental settings are:

1. Pruning: removing unreliable protein interactions from *D* using the AdjustCD method [19,20]. This technique assigns weights to the interactions based on the network topology and considers as unreliable those whose weights are less than a specified threshold. Here, we experimentally set the threshold to 0.2 for weighted datasets and to 0.45 for unweighted datasets.

2. Filtering: identifying bridge, fjord, and shore proteins which could add noise to the network, as defined in [12].

3. Protein Ranking: proteins are ordered using a ranking algorithm, in analogy with the PageRank algorithm.

4. Complex Detection: all the essential proteins, i.e. those that are filtered as noise, are seeds based on which detected complexes are formed using the spoke model. Here, a protein can belong to more than one complex.

5. Pre-processing: The set of predicted complexes is filtered to remove possible duplicates generated due to the introduced overlap assumption.

6. Merging by Cohesiveness: Two detected complexes, whose overlap is above a merging threshold, here 75%, are merged. The subsequent complex is iteratively extended following the presented merging procedure.

7. Post-processing: again, the refined set of predicted complexes is filtered to remove possibly replicated copies of the same complexes resulting from the previous merging step.

### Comparison with other methods

**Figure 6:**
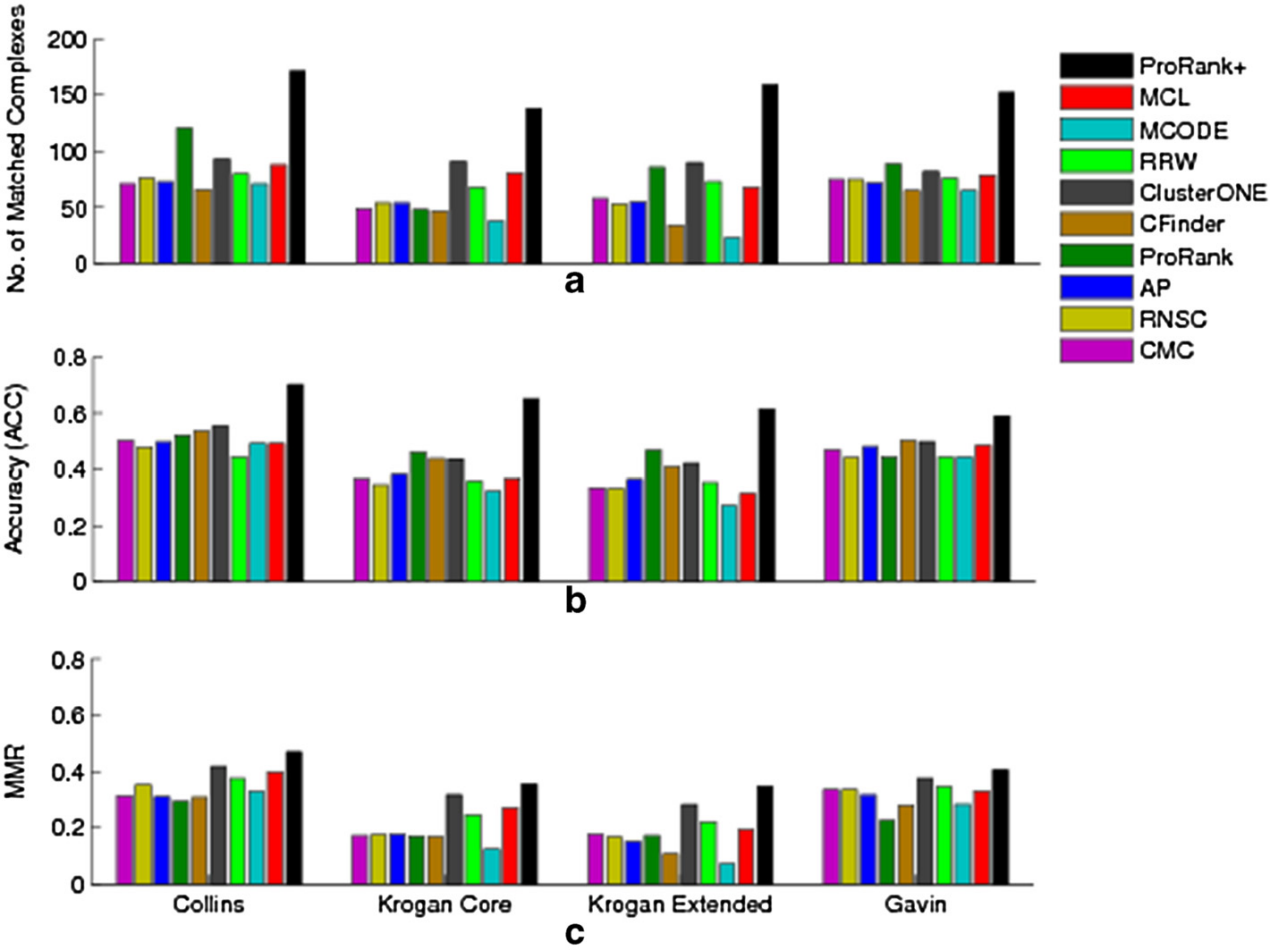
**ProRank + compared to ProRank, MCL, MCODE, CMC, AP, ClusterONE, RNSC, RRW, and CFinder.** Here, the four weighted yeast datasets are used: Collins, Krogan core, Krogan extended and Gavin. The comparisons are in terms of **(a)** the number of clusters that match the reference complexes, **(b)** the geometric accuracy (*Acc*) which reflects the clustering-wise sensitivity (*S*_*n*_) and the clustering-wise positive predictive value (*PPV*), and **(c)** the maximum matching ratio (*MMR*).

**Figure 7:**
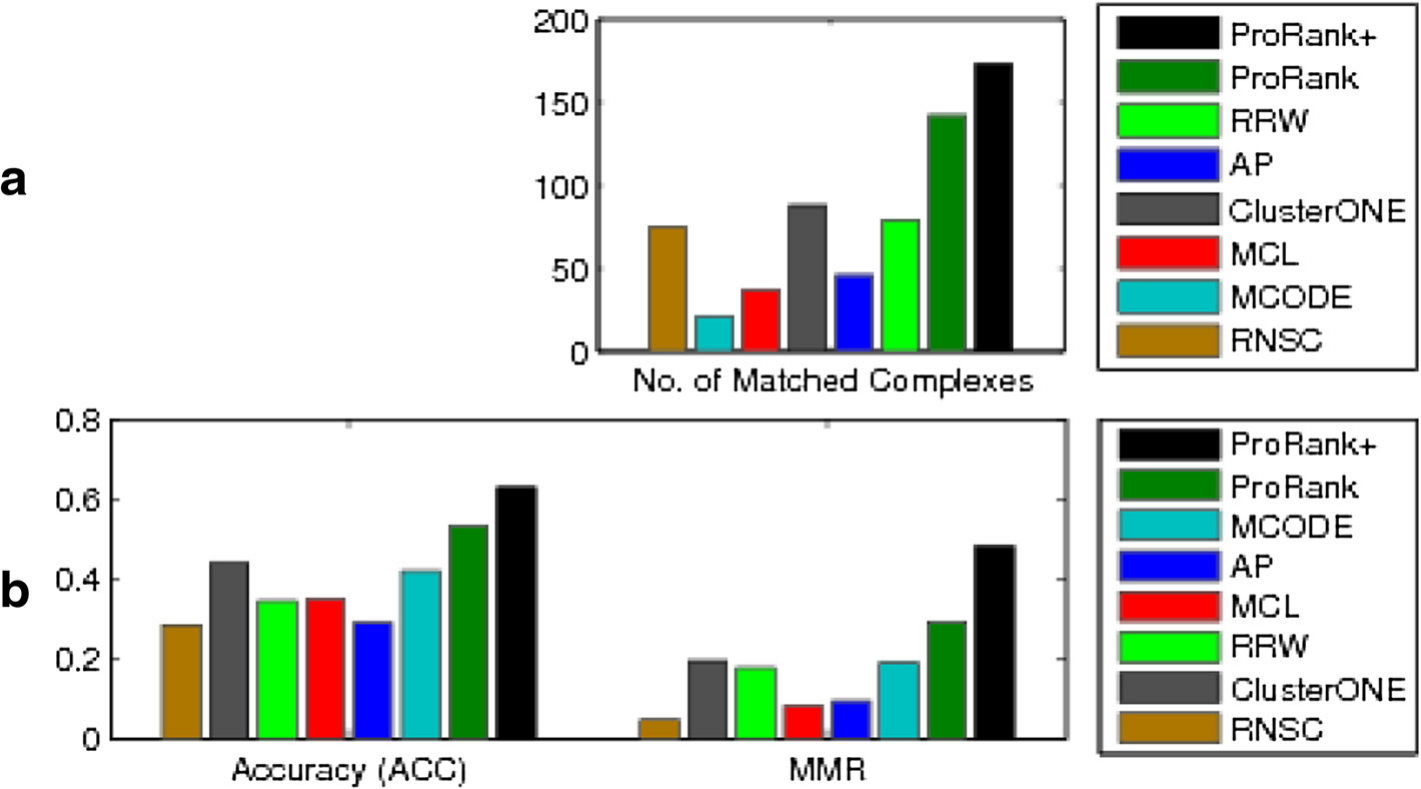
**ProRank + compared to ProRank, MCL, MCODE, AP, ClusterONE, RNSC, and RRW.** Here, the un-weighted BioGRID dataset is used. The comparisons are in terms of **(a)** the number of clusters that match reference complexes, and **(b)** the geometric accuracy (*Acc*) which reflects the clustering-wise sensitivity (*S*_*n*_) and the clustering-wise positive predictive value (*PPV*), and the maximum matching ratio (*MMR*).

ProRank + was compared to other state-of-the-art methods, applied on the same datasets and evaluated based on the same quality scores. These methods include ProRank [12] to highlight the attained improvement, Markov Clustering (MCL) [2], the molecular complex detection (MCODE) algorithm [3], the clustering based on maximal cliques (CMC) method [4], the Affinity Propagation (AP) algorithm [5], ClusterONE [6], the restricted neighborhood search (RNSC) algorithm [7], the RRW algorithm [9], and CFinder [10]. The comparisons among the results scored by these approaches [6] and those scored by ProRank + are displayed in Figures 6 and 7. Since not all the algorithms can be applied to unweighted datasets, fewer methods for instance were applied on the BioGRID dataset.

The experimental results show that ProRank + detected a higher number of protein complexes that matched with the reference set. It is worth-mentioning that the number of clusters predicted by ProRank + is relatively higher than the number of clusters returned by the other methods for Collins, Gavin and BioGRID datasets. Nevertheless, the ratio equivalent to the number of matched complexes over the number of detected clusters falls within the same range of the ratio corresponding to the other methods. Added to that, ProRank + achieved higher clustering-wise sensitivity (*S*_*n*_), geometric accuracy (*Acc*) and maximum matching ratio (*MMR*) for all the considered datasets. However, it could not surpass the clustering-wise positive predictive value (*PPV*) of ProRank which was the highest for all datasets. This can be justified by the fact that *PPV* tends to be lower when the overlaps among the detected complexes are substantial. By the *PPV* formula, a complex-detection algorithm that fully succeeds in detecting the reference complexes has a *PPV* value less than or equals to 1 since there is a matching predicted complex for every reference complex, in addition to other predicted complexes that partially overlap with reference complexes. On the other hand, a dummy detection algorithm which distributes the proteins into separate sets of single elements has a *PPV* value equals to 1, which is greater than the *PPV* of the perfect algorithm that is able to detect all reference complexes. Consequently, *PPV* values must be carefully analyzed since they may not always reflect the competence of a certain method. Moreover, the geometric accuracy (*Acc*) is negatively affected by the predicted complexes that do not match any of the reference complexes. This somehow contradicts the initial purpose of developing methods for the detection of protein complexes which mainly consists of finding previously unknown or undiscovered entities. Accordingly, the *MMR* measure [6] was introduced to overcome such limitations by dividing the total weight of the maximum matching with the number of reference complexes. The *MMR* values achieved by ProRank + are in the favor of the proposed approach. We hereby note that our approach could also be explored using other pruning methods such as the ones introduced in [29,30].

### Testing the ability of ProRank + to detect small complexes

Detecting small protein complexes is not a common feature of complex-detection methods. In fact, it is important to identify such complexes in protein interaction networks. For instance, among the 313 protein complexes included in the MIPS catalogue [22], 104 complexes consist of 2 or 3 proteins (approx. 33%). Most of the approaches which view protein complexes as dense regions in the interaction networks are usually unable to detect complexes of small sizes. In view of that, we also tested the ability of ProRank + to detect small protein complexes. We considered the same yeast datasets that were utilized in the previous experiments. The set consisting of the 104 complexes of small sizes in the MIPS catalogue [22] was formed and used as a reference set. The datasets were filtered by the AdjustCD method with a threshold of 0.2. The corresponding results are shown in Table 2. The table highlights the competency of ProRank + in detecting small protein complexes in terms of the number of matched complexes as well as the accuracy (*Acc*) and the maximum matching ratio (*MMR*) scores.

**Table 2:** Testing ProRank + on small complexes

**Dataset**	**Predicted complexes**	**Matched complexes**	**Sn**	**Acc**	**MMR**
**Collins**	428	91	0.875	0.935	0.433
**Krogan core**	229	34	0.667	0.816	0.163
**Krogan extended**	260	78	0.75	0.769	0.217
**Gavin**	534	57	0.897	0.947	0.293
**BioGRID**	823	78	0.882	0.9	0.351

The results are in terms of (a) the number of clusters that match the reference complexes, (b) the geometric accuracy (*Acc*) which reflects the clustering-wise sensitivity (*S*_*n*_) and the clustering-wise positive predictive value (*PPV*), and (c) the maximum matching ratio (*MMR*).

### Testing ProRank + on Human protein-protein interaction dataset

When tested on various datasets, weighted and unweighted, ProRank + was able to detect more complexes than state-of-the-art methods with higher quality scores. Indeed, the method could be very helpful for biologists if it was tested on Human interactions and proved valuable in detecting known protein complexes of key roles in normal and abnormal cellular functions. Therefore, we applied our method on the Human interactions dataset in the BioGRID repository [26]. The interactions are unweighted, and thus the pruning threshold was set to 0.45. The pruned dataset consisted of 3031 interactions. ProRank + was able to predict 267 protein complexes. We then examined the detected complexes for potential mappings with known protein complexes; some of which are presented in Table 3 and highlighted hereafter.

**Table 3:** Selected complexes detected by ProRank + when tested on human protein-protein interaction dataset

**Detected complex**	**Proteins members of the detected complex**	**Matching percentage**
**CCT micro-complex**	{CCT3, CCT2, CCT8, CCT6A, CCT4, CCT7, CCT5, TCP1}	100%
**Ribosomal protein complex**	{RPL32, RPS17, RPSA, RPL10A, RPL12, SLC25A5, RPL7, RPL18, RPL15, RPL21, RPS6, RPS4X, RPL19, RPL14, RPL4, RPS27L, RPS23, RPS26, RPS16, RPL7A, RPS24, RPS13, RPS15A, RPS8, RPS3A, FAU, RPL11, RPL6, RPL9, RPL5, RPS27, RPL17, RPS2, RPS25, RPS20, NOP56, RPS15, RPL23A, RPS10, RPL10L, RPLP0P6, RPS28, RPS5, RPS9, RPL23, RPL18A, RPS3, RPL37A, RPL31, RPL10, RPL8, RPS11, RPL36, RPS19, RPL30, RPL24, RPS21, RPL27, RPS12, RPL29, RPS29, RPS7, RPL22, RPLP0, RPS14, RPL3, RPLP2, RPL27A, RPL13, RPS18, RPS27A}	81.48%
**PA700-20S-PA28 complex**	{PSMD8, PSMB2, PSMC3, PSMC4, PSMA4, PSMA1, PSMD1, PSMD7, PSMA2, PSMB6, PSMB7, PSMD3, PSMB1, PSMC1, PSMC5, PSMC2, PSMB4, PSMA6, PSMD6, PSMD14, PSMD12, PSMD11, PSMD13, PSMA7, PSMC6, PSMA5, PSMB3, PSMB5, PSMA8, PSMD2}	83.33%
**SWItch/Sucrose NonFermentable (SWI/SNF) complex**	{SMARCA4, SMARCC1, ARID1A, SMARCE1, SMARCC2, SMARCA2, SMARCB1}	60%

1. The CCT micro-complex [31] which participates in protein folding, assembly and transport. It was fully-detected by ProRank + .

2. The Ribosomal protein complex [32] was detected with an 81.48% match. Five additional proteins were detected: SLC25A5, RPS27L, NOP56, RPL10L, and RPLP0P6. Their association with the detected complex may be just noise or, on the contrary, could present biologically meaningful information.

3. The PA700-20S-PA28 complex [33] was detected with a mapping percentage of 83.33%. This complex is a key component of the ATP-dependent proteolytic pathway in eukaryotic cells and is responsible for the degradation of most cellular proteins.

4. A recent publication [34] confirmed that the mutations of the SWItch/Sucrose NonFermentable (SWI/SNF) complex are ubiquitous in various types of cancer. Accordingly, future research efforts will put more focus on this tumor suppressor complex towards better understanding of cancer diseases and in the direction of developing more effective cures. The SWI/SNF complex is composed of ten elements distributed as follows: (a) SMARCA2 or SMARCA4, two mutually-exclusive ATPase enzymatic subunits; (b) ARID1A, ARID1B, or PBRM1, three mutually-exclusive subunits associated to functional specificity; (c) core and accessory subunits including SMARCB1, SMARCC1, SMARCC2, SMARCE1, SMARCD1, SMARCD2, or SMARCD3, PHF10, DPF1, or DPF2, DPF3, and ACTL6A or ACTL6B. We mapped the composition of SWI/SNF with the set of predicted complexes by ProRank+. Our method was able to detect a complex consisting of the elements SMARCA4, SMARCC1, ARID1A, SMARCE1, SMARCC2, SMARCA2, SMARCB1. In comparison with the known structure of SWI/SNF, ProRank + correctly predicted six members out of ten corresponding to 60% of its subunits with a relatively low number of false positives.

The above experiment affirms the ability of ProRank + to identify significant and key protein complexes from protein interaction data. In addition, such outcomes could potentially contain relevant and previously-undiscovered protein complexes or unidentified protein members of certain complexes.

## Conclusions

In this paper, we presented ProRank+, an efficient method for detecting protein complexes in protein-protein interaction networks. The detection process is mainly centered on a ranking algorithm that allows the identification of key proteins based on which the corresponding components are formed. It is also tailored by a series of pruning, filtering and merging steps, allowing the refinement of the drawn complexes. Unlike most approaches, the design of our method is not bound by the sole association of protein complexes to dense regions in interaction networks. In addition, ProRank + takes into account possible overlaps among complexes and this is an important assumption that reflects biological facts. In contrast with other methods, the experimental study underlined the competitive ability of ProRank + to identify protein complexes. The performance of our algorithm was tested using weighted and un-weighted datasets, and using Human protein interaction data as well. The results were in favor of the introduced approach.

## Competing interests

The authors declare that they have no competing interests.

## Authors’ contributions

EMH and NZ have contributed to the conceptual development of the method. EMH has performed the experimental work and the statistical analysis. Both authors contributed to the manuscript writing. All authors read and approved the final manuscript.
